# Cardiac Transthyretin Amyloidosis With Coincident Paget’s Disease: A Case Report

**DOI:** 10.7759/cureus.42621

**Published:** 2023-07-28

**Authors:** Mohmmad H ALQattan, Mukhtar A Alqadhi, Abdullah A AlKhamis, Ali M Alawadh, Abdulmajeed M Al Omair

**Affiliations:** 1 Nuclear Medicine, Al-Ahsa Health Cluster, Al-Ahsa, SAU; 2 Radiology, Al-Ahsa Health Cluster, Al-Ahsa, SAU

**Keywords:** attr, transthyretin amyloidosis, paget’s disease, amyloidosis, attr cardiac amyloidosis

## Abstract

Cardiac amyloidosis is a condition that results from the accumulation of amyloid proteins in the extracellular matrix of the myocardium. The diagnosis of this disease was challenging as it lacked distinct clinical symptoms and required a biopsy to confirm amyloid deposition. However, there is increasing evidence of non-invasive diagnostic criteria for cardiac amyloidosis, especially for the transthyretin (TTR) type. We report a case of a patient with both cardiac transthyretin amyloidosis (ATTR) and Paget's disease, and we highlight the various radiological features of these two conditions using hybrid imaging techniques. In addition, we discuss the diagnostic imaging characteristics of ATTR cardiac amyloidosis.

## Introduction

Amyloidosis is a group of uncommon conditions that result from the buildup of extracellular deposits of misfolded proteins as insoluble fibrils, leading to a gradual decline in the function of multiple organs and ultimately death [1-2]. The gold standard method for accurately diagnosing cardiac amyloidosis is an endomyocardial biopsy [3]. Advancements in cardiac imaging, diagnostic techniques, and treatments have enhanced the identification and management of cardiac amyloidosis in recent days [2]. Although recent advancements have been made in the diagnosis and management of cardiac amyloidosis, the condition is still frequently undiagnosed, especially in elderly patients [4].

The two most prevalent types of cardiac amyloidosis are immunoglobulin light chain (AL) and transthyretin (ATTR) amyloidosis [5]. Non-invasive diagnostic criteria have been established for the diagnosis of ATTR amyloidosis [2]. In patients with typical echocardiographic or cardiac magnetic resonance imaging (MRI) findings, the diagnosis of ATTR cardiac amyloidosis can be made without the need for histology, provided that 99mTc-pyrophosphate (PYP), 99mTc-3,3-diphosphono-1,2-propanodicarboxylic acid (DPD), or 99mTc-hydroxymethylene diphosphonate (HMDP) scintigraphy shows grade 2 or 3 myocardial uptakes of the radiotracer, and clonal dyscrasia is excluded by serum free light-chain (FLC) assay, serum protein immunoelectrophoresis (SPIE), and urine protein electrophoresis with immunofixation (UPIE) tests [6]. In this case, we emphasize the various approaches recommended for the diagnosis of ATTR cardiac amyloidosis, from initial clinical suspicion to the ultimate confirmation using non-invasive diagnostic techniques.

## Case presentation

A 68-year-old male Saudi presented to the emergency department with complaints of shortness of breath upon exertion, chest pain, and severe lower limb edema bilaterally. He had a known medical history of diabetes mellitus, hypertension, and benign prostatic hypertrophy (BPH). The patient did not present with any other clinical features that could raise suspicions of amyloidosis, such as peripheral neuropathy, autonomic dysregulation, or carpal tunnel syndrome. The patient’s vital signs are tabulated in Table 1.

**Table 1:** The patient's vital signs on presentation

Vital signs	Value	References
Blood pressure	146/81 mmHg	Systolic <120 mmHg; diastolic <80 mmHg
Heart rate	82 beats/min	60-100 beats/min
Respiratory rate	20 breaths/min	12-18 breaths/min
Oxygen saturation	98% on room air	95%-100%

**Figure 1:**
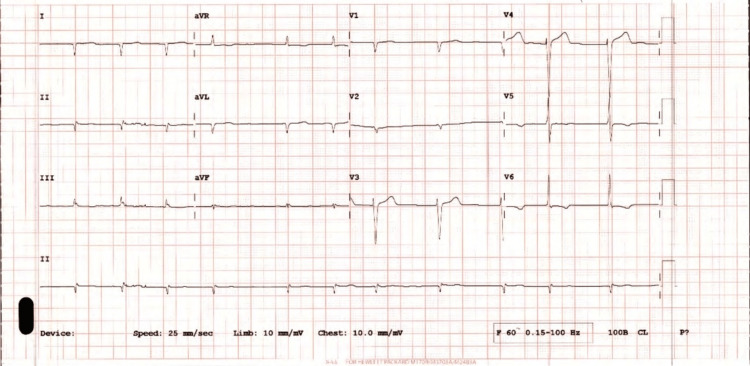
The patient's electrocardiogram showed atrial fibrillation and low QRS voltage

The electrocardiogram (ECG) showed atrial fibrillation and low QRS voltage. There were no ECG features of acute myocardial infarction or ischemia (Figure 1).

The patient's laboratory workup is summarized in Table 2.

**Table 2:** The patient's laboratory workup l: low; h: high

Lab	Value	References
White blood cells	4.12	4-10 x 10^9/l
Hemoglobin	9.6 l	13-17 g/dl
Platelet count	134	130-400 x 10^9/l
Glucose (Random)	6.517	5.4-7.6 mmol/l
Urea (Blood urea nitrogen)	5.3	3.2-7.1 mmol/l
Creatinine	68.8	53-120 umol/l
Calcium, total	2.18	2.1-2.55 mmol/l
Sodium serum	141	137-145 mmol/l
Potassium serum	4.2	3.5-5.1 mmol/l
Chloride serum	102	98-107 mmol/l
Alanine aminotransferase	19	30-65 u/l
Aspartate aminotransferase	27	15-46 u/l
Total protein serum	64.6	63-82 g/l
Albumin serum	36.5	35-50 g/l
Alkaline phosphatase, serum	141 h	38-126 u/l
Troponin-T	0.017	<0.017ug/l

**Figure 2:**
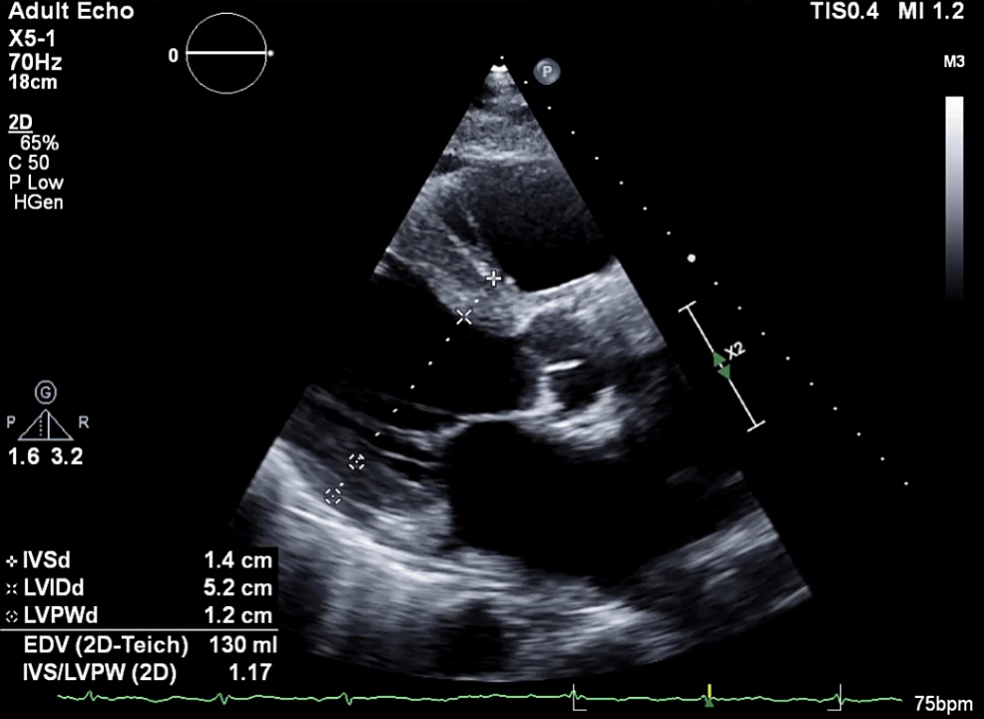
A parasternal long-axis view of an echocardiogram indicated moderate concentric left ventricular hypertrophy with a posterior wall thickness of 12 mm and a thickened interventricular septum measuring 14 mm IVSd: interventricular septal end diastole thickness; LVIDd: left ventricular internal diameter end diastole; LVPWd: left ventricular posterior wall end diastole thickness; EDV: end-diastolic volume; IVS/LVPW: the ratio of interventricular septum thickness to left ventricle posterior wall thickness

A transthoracic echocardiogram (TTE) revealed the presence of concentric moderate left ventricular wall hypertrophy, right ventricular hypertrophy, right and left atrial enlargement, moderate to severe tricuspid regurgitation, and mild mitral regurgitation (Figure 2).

The estimated left ventricular ejection fraction was 55%.

**Figure 3:**
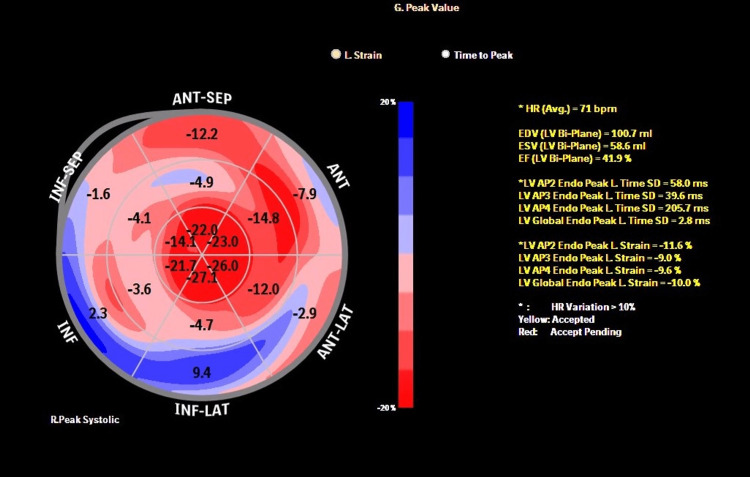
The bull's eye graph of the left ventricle displayed a "cherry on top" or an apical sparing pattern, revealing relative preservation of longitudinal strain at the apex

The global longitudinal strain (GLS) revealed preserved apical with decreased basal and mid-segment values of the left ventricular wall in a "cherry on top pattern," suggesting cardiac amyloidosis (Figure 3).

As a result, the patient underwent diagnostic testing for amyloid cardiomyopathy. Serum and urine immunoglobulin electrophoresis did not show any abnormal bands, and the light chain assay was normal. A bone marrow aspiration did not reveal any amyloid deposits.

**Figure 4:**
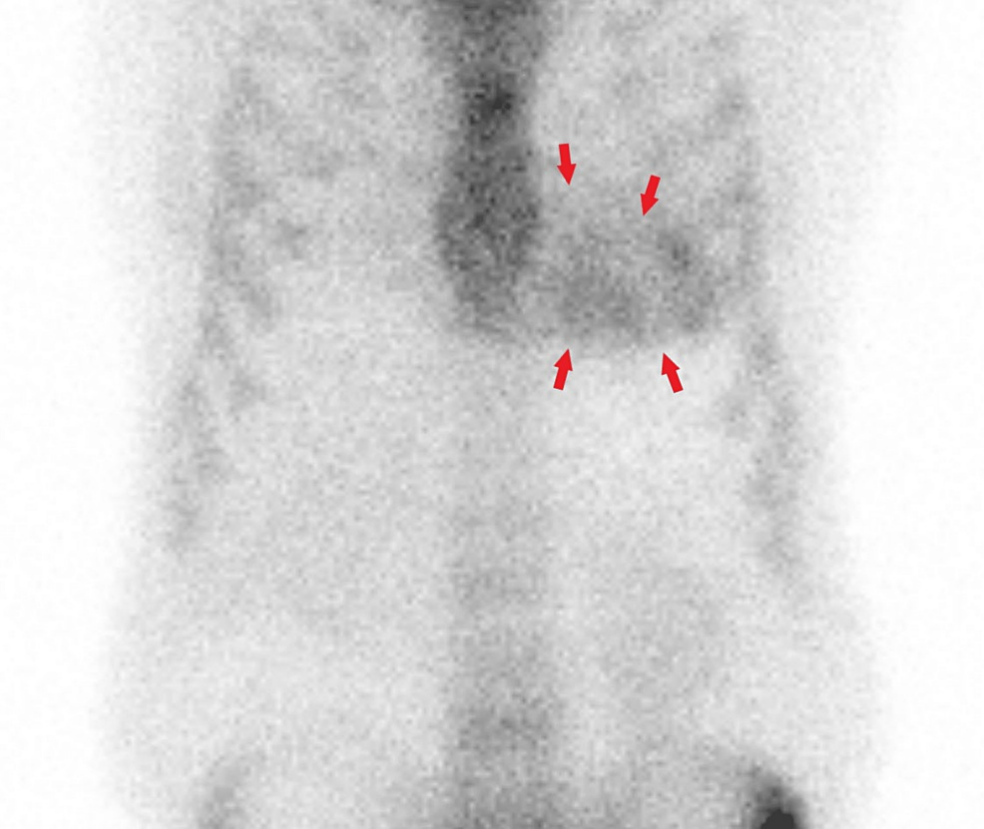
A three-hour delayed static anterior planar image demonstrated heterogeneous diffuse myocardial uptake (arrows) similar to the adjacent ribs

Radionuclide scintigraphy with three-hour delayed 99m-technetium-labeled HMDP static chest images revealed a myocardial uptake similar to that of adjacent bones (Figure 4).

**Figure 5:**
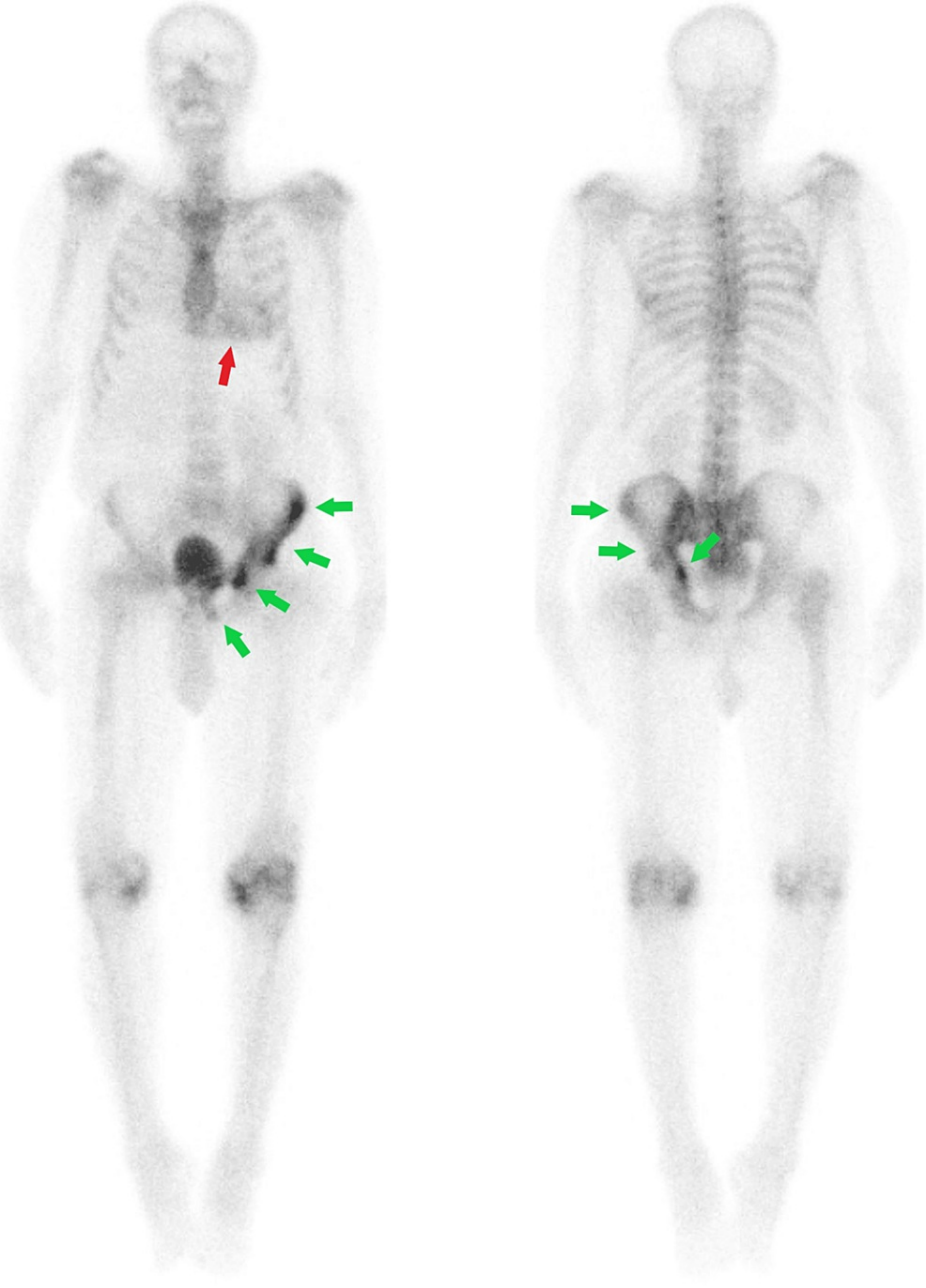
The three-hour delayed anterior and posterior whole-body images demonstrated diffuse heterogeneous myocardial uptake (red arrow) with markedly increased radiotracer uptake involving the left hemipelvis (green arrows)

**Figure 6:**
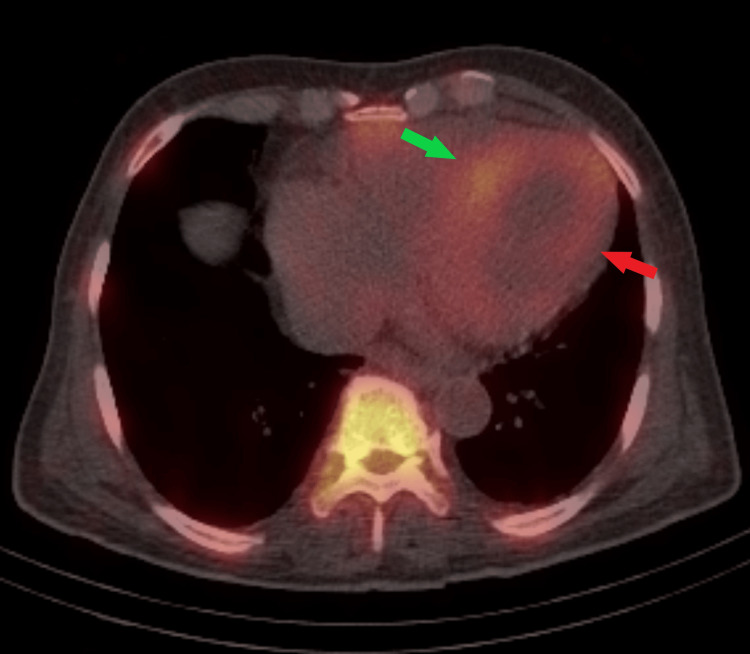
Fused SPECT-CT images of the chest demonstrated diffuse left ventricular myocardial uptake (red arrow) with a focal increase on top of diffuse increased uptake localized to the interventricular septum (green arrow), which appeared similar to the ribs uptake

Then, a whole-body planar and chest single-photon emission computed tomography (SPECT) scan with computed tomography (SPECT-CT) images were performed, which demonstrated diffuse heterogeneous left ventricular myocardial uptake that appeared similar to the ribs uptake, consistent with a grade 2 ATTR amyloidosis on semiquantitative assessment (Figures 5-6), respectively.

**Figure 7:**
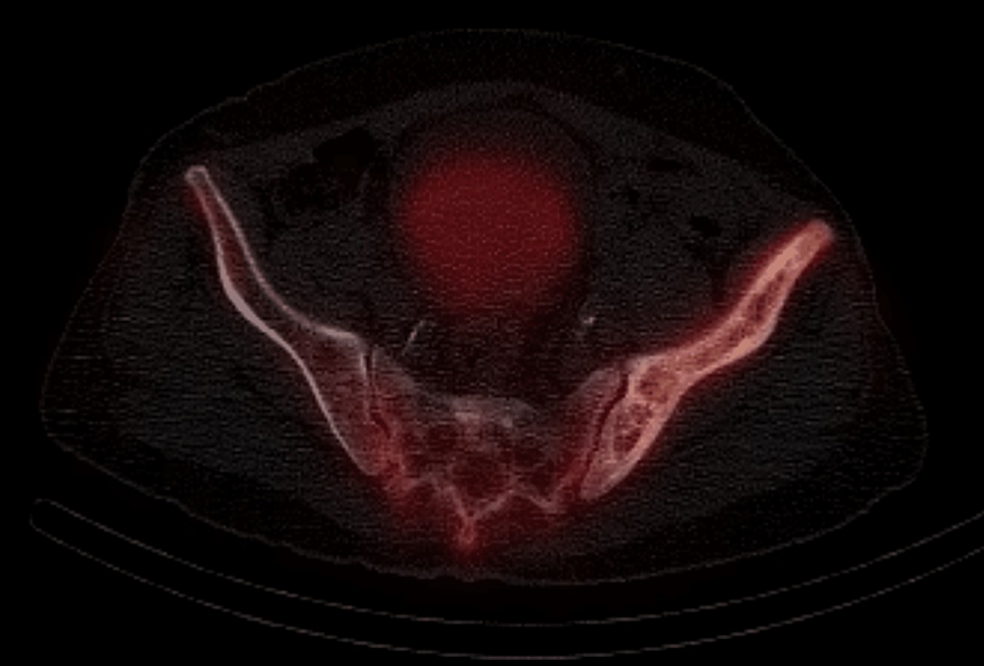
Selected axial SPECT-CT images showed heterogeneous sclerosis, cortical thickening, and mild bone expansion of the left hemipelvis with moderate to marked increases in radiotracer uptake

Also, there was an incidental finding of diffuse heterogeneously increased radiotracer uptake involving the left iliac, left superior and inferior pubic rami, as well as the left ischium, which corresponds to heterogeneous sclerosis, cortical thickening, and mild bone expansion on SPECT-CT images (Figure 7).

Overall radiological features, along with abnormally high alkaline phosphatase, are highly suggestive of Paget’s disease.

**Figure 8:**
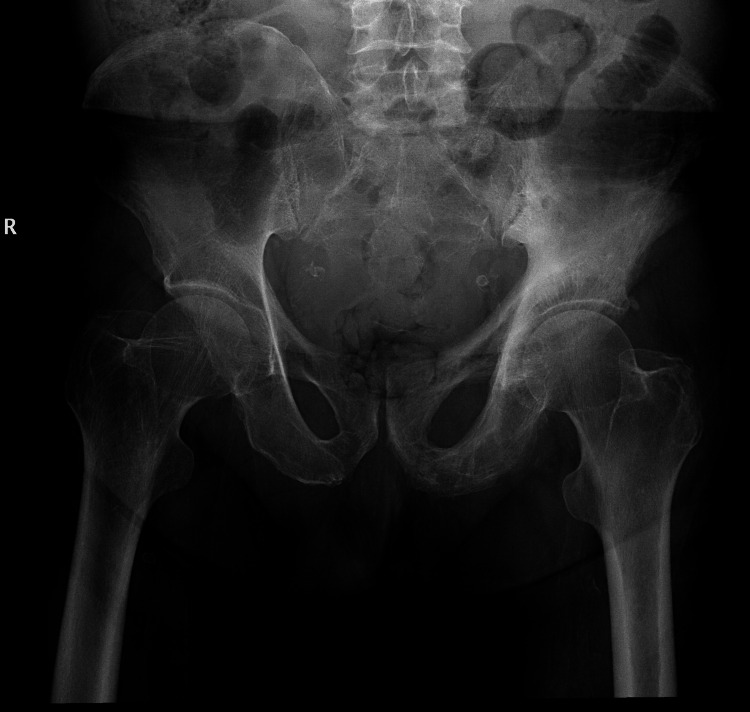
A plain anterior-posterior (AP) radiograph of the pelvis demonstrated coarse bone trabeculation, sclerosis, and bone expansion of the left hemipelvis, with thickening of the left iliopectineal and ilioischial lines

A complimentary pelvic radiograph was performed, which concurred with the CT component findings and our initial interpretation of Paget’s disease (Figure 8).

The patient was treated for congestive heart failure, atrial fibrillation, and hypertension as an inpatient. He was discharged after stabilization with medications such as furosemide, indapamide, valsartan, bisoprolol, esomeprazole, apixaban, rosuvastatin, omnic, and ferrous sulfate. A low-fat, low-salt diet was also recommended. Tafamidis, a disease-modifying agent for ATTR, has been requested and is awaiting administrative approval. Since the patient was asymptomatic for Paget’s disease, the treating team decided to keep the patient under follow-up without treatment for the time being. On the follow-up visit, the patient was stable and asymptomatic.

## Discussion

Amyloid fibril deposition in the extracellular matrix causes cardiac amyloidosis. The diagnostic approach for this condition varies based on the clinical presentation and the outcomes of initial testing [7]. Typically, clinical features such as proteinuria (even in mild cases), macroglossia, skin bruises, carpal tunnel syndrome, and heart failure are suggestive of cardiac amyloidosis [2].

Electrocardiogram changes may include low QRS voltage, left bundle branch block, conduction abnormalities, and atrial fibrillation [7]. Atrial fibrillation is frequently observed in patients with cardiac amyloidosis and is more prevalent in those with ATTR amyloidosis compared to AL amyloidosis [8].

Echocardiography is a crucial diagnostic tool for cardiac amyloidosis. The most frequent phenotype observed is increased thickness of the left ventricular wall, along with atrial enlargement and indications of elevated filling pressures resulting from restrictive diastolic filling. Other signs such as right ventricular hypertrophy, pericardial effusion, and thickening of the atrial septum or cardiac valves may be observed. The presence of relative apical sparing of longitudinal strain (LS) is a notable characteristic of cardiac amyloidosis [9].

It is essential to perform serum and urine protein electrophoresis and immunofixation to detect monoclonal proteins in patients displaying echocardiographic or CMR findings consistent with amyloidosis. The absence of monoclonal proteins can assist in confirming the diagnosis of ATTR amyloidosis [10].

Cardiac scintigraphy using 99mTc-labeled tracers for the diagnosis of ATTR cardiac amyloidosis has been well-established [11].

The Japanese Circulation Society (JCS) guidelines for the diagnosis and treatment of cardiac amyloidosis, as well as expert consensus recommendations from the American Society of Nuclear Cardiology (ASNC), Amyloidosis Research Consortium, and European Society of Cardiology, now incorporate 99mTc-labeled bone radiotracer scintigraphy into their diagnostic algorithms [12].

Bone tracer cardiac scintigraphy, which uses 99m technetium (Tc)-labeled DPD, pyrophosphate (PYP), and HMDP, can aid in the identification and diagnosis of ATTR cardiac amyloidosis [11]. The Perugini staging system is used to grade the scintigraphy findings based on visual scoring, with grade 0 indicating no myocardial uptake and normal rib uptake, grade 1 indicating myocardial uptake less than adjacent rib uptake, grade 2 indicating myocardial uptake equal to rib uptake, and grade 3 indicating myocardial uptake greater than rib uptake [2]. ATTR cardiac amyloidosis typically exhibits grade 2 or 3 uptakes as it is highly attracted to bone tracers [2]. In contrast, AL amyloidosis usually demonstrates absent or grade 1 myocardial uptake [13].

The imaging protocol for 99mTc-PYP/DPD/HMDP based on ASNC guidelines recommends two- to three-hour SPECT and planar imaging obtained after injection of the radiotracer with anterior and lateral views of the chest or heart region. Optional whole-body planar imaging may be considered to evaluate for shoulder or hip joint involvement with systemic amyloidosis [14]. In our institute, whole-body planar imaging is part of the routine protocol for bone tracer cardiac scintigraphy.

**Figure 9:**
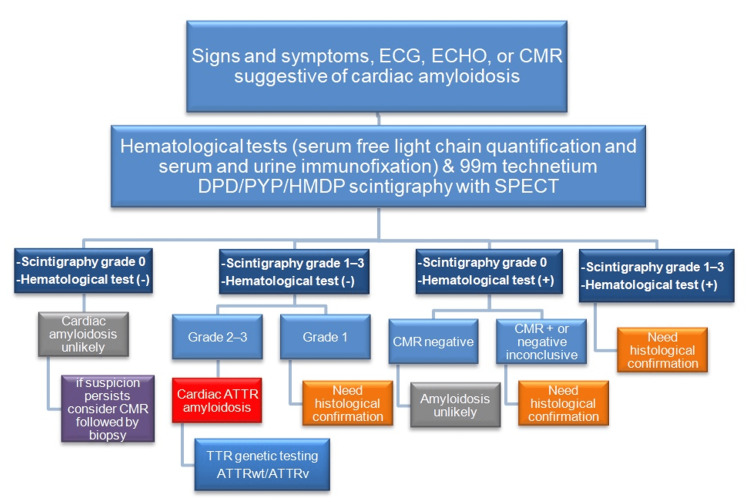
A diagnostic algorithm for cardiac amyloidosis ECG: electrocardiogram; ECHO: echocardiography; CMR: cardiac magnetic resonance; SPECT: single photon emission computed tomography; ATTR: transthyretin amyloidosis; ATTRwt: wild-type transthyretin amyloidosis; ATTRv: hereditary transthyretin amyloidosis.

The European Society of Cardiology 2021 proposes a diagnostic algorithm focusing on identifying these subtypes by the initial use of 99mTc-PYP, DPD, or HMDP scintigraphy whenever cardiac amyloidosis is suspected (Figure 9) [2].

The treatment of cardiac amyloidosis primarily involves the management of heart failure and other cardiac complications, as well as disease-modifying therapy [13].

**Figure 10:**
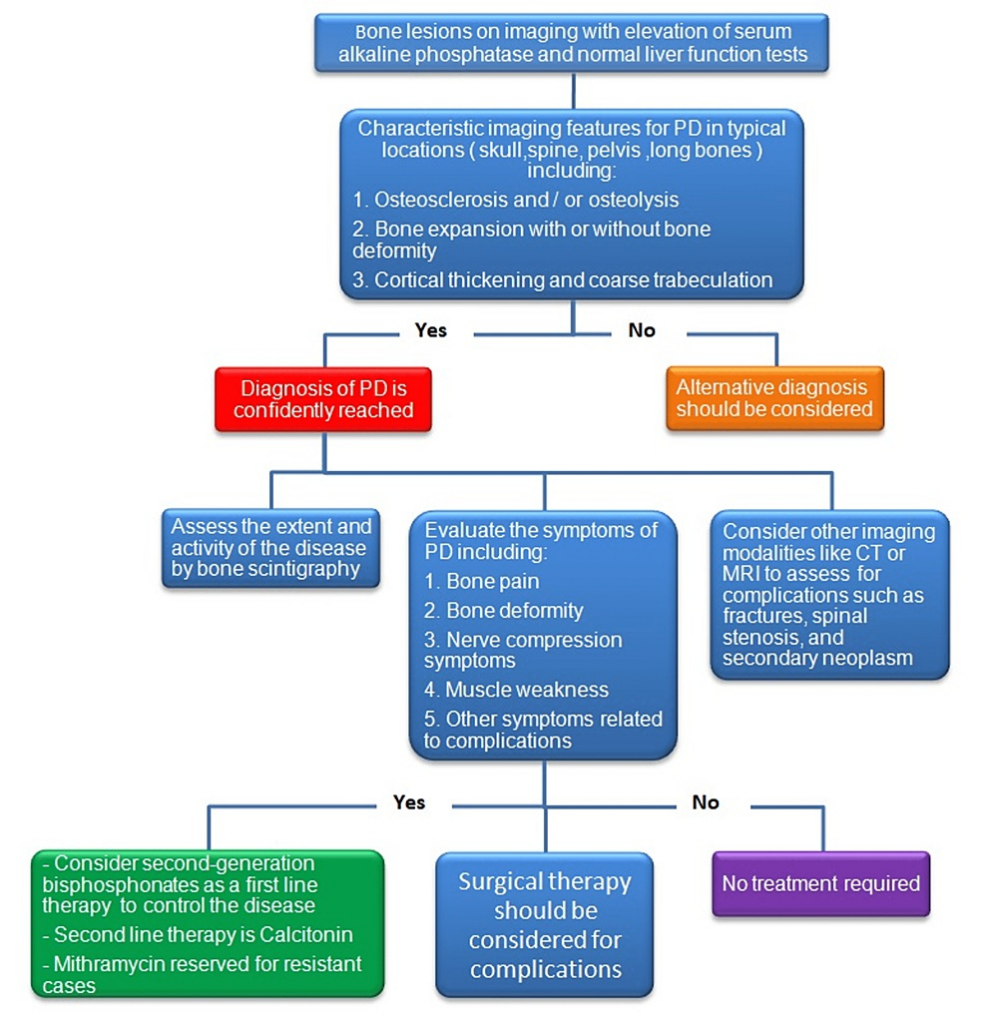
Diagnostic and management algorithm for Paget’s disease PD: Paget’s disease; CT: computed tomography; MRI: magnetic resonance imaging

Paget's disease is the second most common bone disease in the elderly in the United States and is characterized by excessive abnormal bone remodeling [15-16]. About 20% of Paget's disease patients are asymptomatic and are diagnosed incidentally by radiological studies performed for other reasons [15]. The laboratory finding of increased serum alkaline phosphatase in combination with characteristic radiological features is diagnostic for Paget’s disease; the management of Paget’s disease mainly depends upon the clinical presentation and whether associated complications are present or not [15]. We propose an algorithm that summarizes the diagnostic and management approaches for Paget’s disease (Figure 10).

To the best of our knowledge, no apparent association between cardiac amyloidosis and Paget's disease has been identified.

## Conclusions

Amyloid fibril deposition in the extracellular matrix causes cardiac amyloidosis. The diagnostic approach for this condition varies based on the clinical presentation and the outcomes of initial testing. The main goal of this case presentation is to highlight the importance of this disease entity, which seems to be underdiagnosed. Also, it is important to discuss the non-invasive diagnostic features of the disease, as these are well-established for the diagnosis of ATTR-type cardiac amyloidosis and usually prevent other invasive options such as endomyocardial tissue biopsy.
